# Hydroxyurea and blood transfusion therapy for Sickle cell disease in South Asia: inconsistent treatment of a neglected disease

**DOI:** 10.1186/s13023-021-01781-w

**Published:** 2021-03-23

**Authors:** Thamal Darshana, David Rees, Anuja Premawardhena

**Affiliations:** 1 Department of Medical Laboratory Sciences, Faculty of Allied Health Sciences, University of Sri Jayewardenepura, Gangodawila, 10250 Nugegoda Sri Lanka; 2 King’s College Hospital, London, UK; 3 Department of Medicine, University of Kelaniya, Kelaniya, Sri Lanka

**Keywords:** Sickle cell, South Asia, Hydroxyurea, Blood transfusion

## Abstract

**Background:**

Hydroxyurea and blood transfusion therapies remain the main therapeutic strategies for Sickle cell disease. Preliminary data suggest substantial variation and inconsistencies in practice of these two therapeutic modalities in South Asia. In this systematic review we searched Medline, Cochrane library and Scopus for articles on usage of hydroxyurea and blood transfusion therapies for sickle cell disease in South Asia published in English between October 2005 and October 2020.

**Results:**

We selected 41 papers: 33 from India, 3 from Sri Lanka, 2 each from Pakistan and Bangladesh and one from Nepal. Only 14 prospective trials focused on hydroxyurea therapy from which majority (n = 10; 71.4%) adopted fixed low dose (10 mg/kg/day) regimen. With hydroxyurea therapy, 12 and 9 studies reported significant reductions in vaso-occlusive crises and transfusion requirement respectively. Severe anaemia (haemoglobin level < 6–7 g/dl) was the commonest indicator (n = 8) for transfusion therapy followed by vaso-occlusive crisis.

**Conclusions:**

Published data on the hydroxyurea and transfusion therapies in South Asia are limited and heterogeneous. A clear gap of knowledge exists about the nature of the sickle cell disease in the Indian subcontinent particularly from countries outside India necessitating further evidence-based assessments and interventions.

## Background

Sickle cell disease (SCD) is the commonest monogenic disorder characterized by a single mutation in the gene encoding for β-globin chain (HBB). The prevalence of the disease is high in sub-Saharan region of Africa, parts of Mediterranean, India and in the Middle East [1]. Remarkable variability of the clinical severity of SCD is widely acknowledged. The phenotypic variability could extend from those with very mild disease where patients may lead life without any need for treatment to individuals with severe complicated disease with multiple disabling symptoms leading to premature death [2]. Five classical HBB haplotypes of SCD (Central African Republic, Benin, Senegal, Cameroon and Arab-Indian) have also described and are widely believed to contribute to the phenotypic variability largely through their effects on foetal haemoglobin (Hb F) levels [3]. Irrespective of the haplotype, evidence suggests that poverty influenced by lower socio-economic status could lead to adverse outcomes in the disease [4, 5]. In the western world, more than 90% of children with SCD survive to adulthood whilst in Sub-Saharan Africa where there is the greatest burden of sickle cell anaemia estimates suggest that 50–80% of patients will die before adulthood [2].

In South Asia, the highest prevalence of the SCD is observed in India, where over 20 million patients with the disease are known to live. The burden of the disease in India is estimated to be second only to that of Africa with the highest frequency of βs allele being found in a belt stretching across central India, from South-eastern Gujarat to South-western Odisha [6]. Although SCD has been reported from Pakistan, Sri Lanka, Nepal, Bangladesh and the Maldives, very little is known about the nature of SCD and the burden of the disease in these countries. There are few survival and mortality studies from South Asia. In a study conducted in Gujarat in India, about 20% of children with SCD died by age of two and 30% of children with SCD from tribal areas were noted to die before they come reached adulthood [7].

Several preventive and treatment approaches are available for management of SCD. Though no all-encompassing single guideline for management of SCD exists, expert committees have developed several guidelines on trial-based evidence and best practices [8–10]. These guidelines largely do not take into consideration the genotype nor the locality in which the patient is being treated.

Currently available treatment options for SCD include using of disease-modifying therapies like hydroxyurea (HU), blood transfusion and for a very few patients using near curative treatments like hematopoietic stem cell transplantation, and gene therapy. Supportive and preventive strategies like daily oral prophylactic penicillin up to the age of 5 years, opioid therapy to relief acute pain related to sickling event, non-opioid analgesics for chronic pain related to sickling and yearly Trans Cranial Doppler (TCD) examination from the ages 2–16 years to identify those who are vulnerable for stroke form the back bone of any management strategy [8, 11]. In addition, voxeloter, L-glutamine and crizanlizumab have all been approved by U.S. Food and Drug Administration (FDA) recently, and are likely to expand the future therapeutics option for SCD [12].

The two main strategies of SCD management, namely HU and blood transfusion are used based on specific requirements. Blood transfusion therapy has been used for patients with SCD expecting that the normal haemoglobin would compensate for the adverse events generated by sickle haemoglobin (Hb S). Acute transfusion is generally performed to prevent / reverse severe anaemia or as an exchange transfusion for immediate reduction of sickle cell related acute complications [13]. Chronic transfusions are predominantly used for primary stroke prevention, or to prevent the recurrence of stroke among children with SCD, and to reduce recurrent vaso-occlusive crisis (VOC) and acute chest syndrome (ACS) when HU is ineffective [14–16]. HU, a cytotoxic drug, is used in the hope of altering the marrow-proliferation in favour the production of Hb F over Hb S. Evidences suggest usage of HU reduces the incidence of acute pain, rate of acute chest syndrome, blood transfusion and overall mortality among patients with SCD [17, 18]. Furthermore, HU decreases the numbers of platelets and white cells reducing harmful effects interceded by them in vascular injuries [19].

Preliminary literature assessment suggested that the management of SCD in South Asian countries appears to vary and is not consistent with the generally practiced guidelines for SCD [20, 21]. We decided to conduct this review on the two main modalities of SCD treatment, namely blood transfusion and HU therapy across the different countries in South Asia to see how its applied in this region.

## Methods

### Search strategy

We searched databases of MEDLINE via Pubmed, Cochrane library (CENTRAL) and Scopus by Elsevier for studies published in English for past 15 years (between October 2005 and October 2020) using the following keywords in many combinations: Sickle cell, Sickle cell anaemia, Sickle cell disease, Blood transfusion, Hydroxycarbamide, Hydroxyurea, South Asia, India, Pakistan, Sri Lanka, Bangladesh, Nepal, Bhutan and Maldives.

### Inclusion criteria

Prospective trials, descriptive studies, randomized placebo-controlled trials, reviews and case series reporting the practice of blood transfusion and Hydroxyurea therapies for SCD in seven South Asian countries (India, Pakistan, Sri Lanka, Bangladesh, Nepal, Bhutan and Maldives) were included in the present review.

### Exclusion criteria

SCD related Studies that did not describe the practice of blood transfusion and Hydroxyurea therapies for SCD in aforementioned seven South Asian countries were excluded. Also, studies which were non-peer reviewed, unpublished and duplicate of a previously included study were excluded from the present review.

### Data extraction

Two researchers (T.D. and A.P.) independently reviewed all abstracts of journal articles gathered by web search to identify papers that required full-text review. Final decision of selection was made via consensus. Furthermore, all articles were discussed with a third independent reviewer (D.R.). Data on the study setting, objectives, methods and results of each selected articles were extracted. Moreover, we methodically searched for any related papers in the reference lists of all articles selected.

## Results

**Fig. 1:**
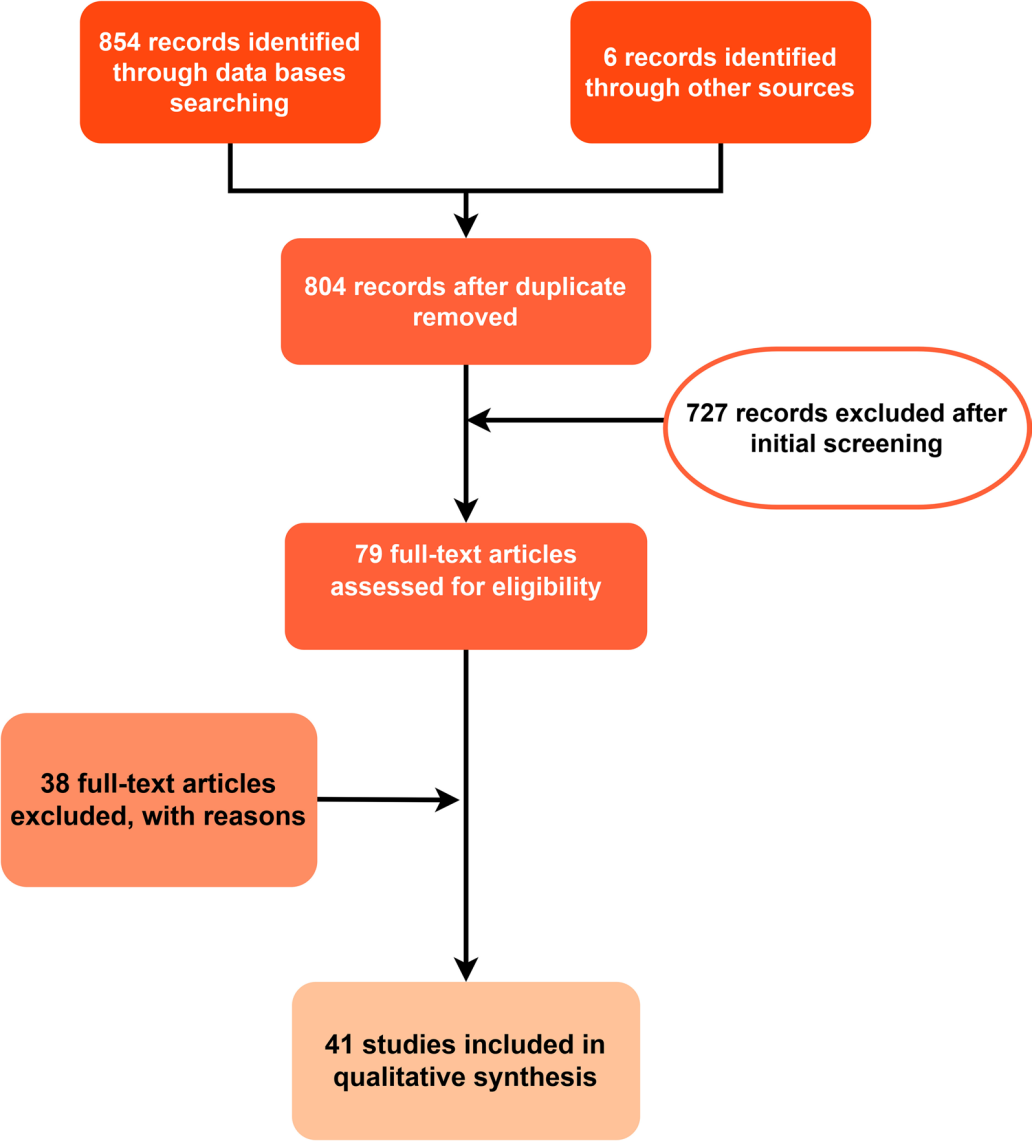
Flow of information through different phases of the systematic review

**Fig. 2:**
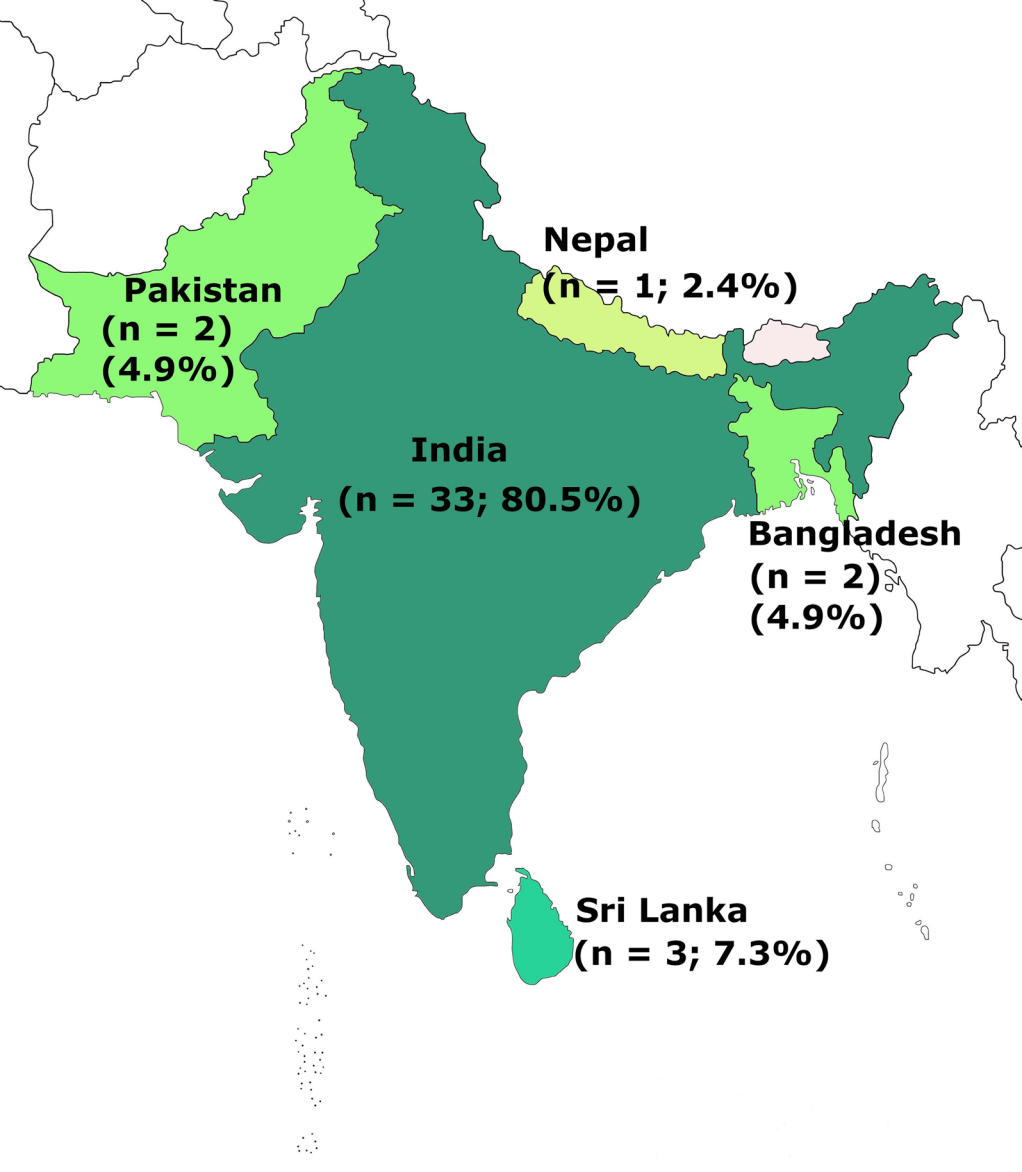
Frequencies of data coming from each South Asian country

We identified 860 papers through the search strategy, of which only 41 articles were in compliance with inclusion criteria were selected for qualitative synthesis (Fig. 1). Out of the 41 articles 33 (80.5%) originated from India. In addition, there were 8 eligible papers including, 3 (7.3%) from Sri Lanka and 2 each (4.9%) from Pakistan, Bangladesh and one from Nepal (Fig. 2). Among the 8 studies selected outside India in South Asia, there were 7 case reports with 2 case studies each from Pakistan, Sri Lanka, Bangladesh and one from Nepal [22–28]. No eligible study was identified from Maldives and Bhutan. The majority of articles (58.5%; n = 24) were published during the last 5 years (2015–2020). Designs of the 34 studies excluding case reports included prospective cohort studies (n = 15), descriptive studies (n = 7), retrospective analyses (n = 5), reviews (n = 3), prospective cohort comparison (n = 2), analytical cross-sectional (n = 1) and randomized placebo-controlled trial (n = 1).

Of the 34 studies excluding case reports (Table 1), 14 Indian studies (41.2%) focused on HU therapy while 6 studies (17.6%) focused on transfusion therapy. Seven studies (20.6%) were descriptive studies of clinical characteristics and 4 were observational cohort studies. Furthermore, 3 reviews described the clinical manifestation of SCD and the role of hydroxyurea in sickle patients with Asian haplotype. Of the 14 prospective Indian studies focused on HU therapy, 12 (85.7%) and 9 (64.3%) studies noted significant reductions in VOC and blood transfusion requirement respectively with HU therapy. Likewise, 5 (35.7%) studies reported significant reduction in hospitalization and 2 (14.3%) studies noted significant improvement in ACS following the HU therapy. Of the 14 prospective studies focused on HU therapy, 13 analysed the haematological profiles of the participants. Significant improvement, particularly in haemoglobin level and Hb F level was observed in the majority (n = 13; 100.0%, n = 10; 76.9% respectively). Of the 13 studies, 10 (76.9%) noted significant reduction in WBC with mild-moderate neutropenia prompted by HU therapy. Deshpande et al., analysed the variation of WBC over different age groups and found HU induced leukopenia was significant only amidst SCD children below 10 years of age [20]. However, none of the studies observed severe neutropenia (neutrophil count < 0.5 × 10^9^/L) among the users of HU. Of the 13 studies that analysed haematological profile among HU users, 7 (53.8%) reported significant reduction in platelet count with no severe case of thrombocytopenia (platelet count < 50 × 10^9^/L). Variations were identified in the dose of HU given to patients with SCD. Eleven out of 14 prospective trials adopted fixed dose method and the remaining 3 trials adopted dose escalation regimen of HU. Of the studies adopted fixed dose method; 10 used low dose (10 mg/kg/day) HU regimen and one adopted standard moderate dose (20 mg/kg/day) regimen. Of the studies which adopted dose escalation method, 2 started with low dose and increase up to high dose and one started with the standard moderate dose (20 mg/kg/day) and escalated by 5 mg/kg/day as adjudged by the treating clinician.

**Table 1:** Summary description of the selected studies excluding case reports

Reference	Study design	Related findings	Comments
Italia et al. [29]	A prospective trial involving adult and paediatric homozygous sickle (Hb SS) and adult Hb S-β thalassaemia patients from Madhya Pradesh and Maharashtra of India	Clinical severity scores were significantly reduced by HU therapy among all groups of patients with SCD (*p* < 0.001). After HU therapy 91% patients had no transfusion requirement	Low dose HU therapy (10–15 mg/kg/day) showed impressive improvement in the clinical condition of Indian patients with SCD
Singh et al. [30]	A prospective study involving SCA patients from Chhattisgarh, India	Number of hospital admissions (*p* 0.03) and rate of crisis per year (*p* 0.008) was significantly reduced with HU therapy	HU therapy significantly improved the acute clinical events related the SCA and increased the time interval between transfusions
Patel et al. [31]	A prospective open label observational study involving SCA patients in Eastern India	Rate of pain crisis in the HU therapy groups (0.5/Y) was significantly reduced than the control groups (4.8/Y) (*p* 0.008). Following HU therapy of 2 Years, 95% (19/20) patients become transfusion independent	Low dose HU therapy with no significant toxicity seems to be a useful treatment for SCA patients in Eastern India
Jain et al. [32]	A double blind randomized controlled trial among SCA children in Central India	Event rates per patient per year for VOC, blood transfusions and hospitalization in HU treated group reduced by 95.0, 94.6 and 93.1%, respectively. Also, Hb and Hb F levels were significantly higher in HU treated group than the placebo	Significant haematological and clinical benefits of fixed low-dose HU (10 mg/kg/day) therapy were observed among Indian SCA patients
Lakhkar et al. [33]	A prospective observational study involving SCA patients from Vidarbha, India	61% of children including SCA children with headache (8%) received 5–10 transfusions/year	Transfusion requirement was high in this particular cohort of SCA children
Jain et al. [34]	A retrospective analysis involving SCA children from central India	Majority (62.0%) of the transfusions were required for SCA children below 5 years of age. Transfusions were mostly given when Hb level drops below 5 g/dl	Clinical picture of the SCA children from central India is severe demanding frequent medical attention
Jain et al. [35]	A prospective longitudinal study involving SCA children from Nagpur, India	Rates of VOC, blood transfusions, sequestration crises, stroke, ACS and rate of hospitalizations were significantly reduced (*p* < 0.001) after 2 years with HU treatment	Low fixed dose HU (10 mg/kg/day) could significantly improve the haematological profile with significant clinical benefits
Mehta et al. [36]	A descriptive study of transfusion practice for patients with SCD at a blood bank in southern Gujarat, India	During the 18-week evaluation period 145 transfusion were reported for 96 patients. Ten patients (10.4%) received transfusions even with pre-transfusion Hb level ≥ 8 g/dl	Transfusions appeared to be widely used among the patients with SCD at the respective centre
Jain et al. [37]	Long term observational follow-up study involving Hb SS patients from central India	After the HU therapy mean number of VOC, ACS, hospitalization and severe anaemia were reduced in Hb SS patients	Long-term low fixed dose HU therapy is efficient in reducing adverse clinical events related to SCD
Oberoi et al. [38]	A retrospective study of Hb S-D Punjab patients from Chandigarh, India	Only 5 out of 10 patients were on HU therapy. 8 out of 10 patients received transfusion including 1 transfusion dependent patient	Encouraging response were noted for HU therapy in Hb S-D Punjab patients
Colah et al. [39]	A review of SCD in India	Low-fixed dose HU therapy reduced acute clinical events among patients with SCD. Transfusion demand was variable among different sickle phenotypes and communities	Low-fixed dose HU therapy is beneficial in ameliorating the severity of Indian SCD
Patel et al. [40]	A prospective cohort study involving Hb S-D Punjab patients from Odisha, India	HU therapy significantly reduced the VOC and rate of transfusions (*p* < 0.0001; 0.0008 respectively) among Hb S-D Punjab patients	Low-fixed dose HU therapy is effective in reducing VOC and transfusion requirement among Indian Hb S-D Punjab patients
Nimgaonkar et al. [41]	A descriptive study of quality of care for patients with SCD from tribal community in Tamil Nadu, India	Median annual cost of hospital visits and HU would account for about 18% of the average income of a tribal family. HU was given freely for all the participating patients	Financial support is required for patients with SCD from low-income communities in order to implement a sustainable comprehensive care system
Dehury et al. [42]	A prospective open label observational study involving Hb S-β+ thalassaemia patients from Odisha, India	After the HU therapy, number of blood transfusion per year, VOC & hospitalization reduced significantly (*p* < 0.0001)	Low fixed dose HU therapy is effective in improving the clinical profile of Hb S-β+ thalassaemia patients
Italia et al. [43]	A descriptive study involving patients with SCD from central and western India	35% and 39.1% of Hb SS and Hb S-β thalassaemia patients who were classified having severe clinical course had 1–5 times transfusions per year	Transfusion requirement appeared to be higher in the more severe SCD than the milder version
Italia et al. [44]	A descriptive study evaluating the feasibility of establishing a new-born screening and follow-up programme for SCD in tribal regions of Gujarat, India	3 out of 32 SCD babies received transfusion for severe anaemia (Hb < 6 g/dl). Only 32 out of 46 SCD babies were responding for follow-up	Even with multiple attempts to engage with a follow-up programme a proportion of affected SCD babies do not respond neglecting standard care
Upadhye et al. [45]	A prospective cohort study involving Hb SS babies from central India	Incidence of blood transfusions was 45.1/100 person years. Babies who experienced several stroke episodes were put on chronic transfusion therapy	Severe anaemia (Hb < 5 g/dl) and history of stroke potentially required blood transfusions
Serjeant [46]	A review discussing a locally appropriate models of care for Indian SCD	Anaemic events are frequent in India. Yet, treatment is often empirical with transfusion without detailed examination	The role of transfusion therapy should be defined for Indian patients with SCD
Jain et al. [47]	A descriptive study involving SCD children from Maharashtra, India	Transfusions were marginally more common in Hb S-β thalassaemia patients than in Hb SS patients. All most all hospitalization due to sickle related clinical event resulted in transfusion. Many patients were receiving HU without any documentation of the clinical course	A proper guideline should be developed on transfusion practice and usage of HU for Indian patients with SCD
Jain et al. [48]	A prospective cohort comparison study involving SCD children from Nagpur, India	24 out of 833 SCD children were on regular transfusion during observation. Median age of starting HU was 12.5 years	Systematic implementation of new-born screening, comprehensive care and HU therapy is necessary for Indian patients with SCD
Yadav et al. [49]	A retrospective cohort study involving patients with SCD from Jabalpur, India	36.5% patients did not require any blood transfusion during 14-year follow-up period. 16.3% required ≥ 3 transfusions. Transfusions given only when the Hb level dropped < 6.5 g/dl	More than 1/3 of the cohort from Jabalpur were able to survive without receiving transfusion for 14 years
Deshpande et al. [20]	A single-centre prospective trial involving patients with SCD from Western India	After the HU therapy, significant reductions were noted in number pain episodes, transfusion requirement and hospitalization due to pain crisis among both adults and children with SCD	HU is beneficial in reducing the pain crisis among patients with SCD thereby improve the quality of the life
Desai et al. [50]	A retrospective study involving pregnant women with SCD from Gujarat, India	52.7% of SCD admissions required transfusions and 8.4% admissions had 3 or more transfusions. Blood transfusions were significantly higher among SCD admissions than non SCD admissions (*p* < 0.01)	There is a high risk of adverse outcomes (including transfusions) in SCD pregnancies than non-SCD pregnancies
Mohanty et al. [51]	A hospital based analytical cross-sectional study involving adult Hb SS patients from Cuttack, India	Even with HU therapy 23 out of 208 patients were on regular transfusions (≥ 3 units/year) while further 32 patients were on occasional transfusion (< 3 units/year)	Even with HU therapy demand for transfusions may still persists among Hb SS patients
Sahoo et al. [52]	A hospital-based prospective study involving Hb SS patients from Odisha, India	Low-fixed dose HU therapy was associated with significant reduction in sperm count, motility and normal morphology (*p* < 0.0001)	Alterations in sperm parameters could be appeared even with low dose HU therapy
Sethy et al. [53]	A prospective single centre study involving adult Hb SS patients from Cuttack, India	After 3 years of low fixed dose (10 mg/kg/day) HU therapy; number of VOC per year and rate of blood transfusion became significantly lower (*p* < 0.001)	Low fixed dose HU therapy was useful in reducing the VOC and transfusion need among adult Hb SS Patients
Jain and Mohanty [54]	A review of clinical manifestation of SCD in India	Transfusion requirement is more in Hb S-β thalassaemia. HU therapy was effective in reducing transfusion requirement, pain crisis and hospitalization	Whether same management protocol could be practiced across the whole India is questionable. There is a need to deliver suitable guidelines for management of Indian SCD patients
Jariwala et al. [55]	A retrospective study involving patients with SCD from Gujarat, India	Mean quantity of transfusion was 8.9 units/patient over 8 years. 11% patients developed allo-antibodies	Even with < 10 transfusions allo-immunization occurred in patients with SCD. Prevalence of allo-immunization was higher in SCD than in β-thalassaemia major
Dave et al. [56]	A longitudinal descriptive study of patients with SCD from tribal area of Gujarat, India	SCD comprehensive care programme increased the coverage of HU from 3.5 to 88%. Rate of transfusion reduced significantly after the enrolment with the programme (27.4 vs 17.8 per 100 patient years)	Good quality care can be provided even for the economically deprived remote communities with SCD
Somkuwar et al. [57]	A prospective cohort study involving Hb SS children from Maharashtra, India	After the HU therapy, rate of acute pain crisis and blood transfusion reduced significantly (*p* = 0.001)	Low-fixed dose HU therapy is safe and effective for Indian Hb SS children
Sinha et al. [58]	A descriptive survey which projected the blood and budgetary requirement for haemoglobinopathies in India (2017–2026)	Annual requirement of blood for SCD would increase by 0.99 million units/year. Projected requirement of blood in 2026 was 9.24 million units	Widespread efficient and effective preventive strategies are urgently required to cope with the sharply increasing demand of blood
Jain et al. [59]	A prospective cohort comparison study involving Hb SS patients from Nagpur, India	26 (33%) Hb SS patients received 74 transfusions (mean 2.8 episodes/patient) Pre-transfusion Hb was below 6 g/dl in 67% of patients. Only 4 out of 103 Hb SS patients were treated with HU	Usage of HU was surprisingly lower among Hb SS patients from Nagpur cohort
Darshana et al. [21]	A descriptive cross-sectional study involving patients with SCD from Sri Lanka	33% (3) of Hb SS patients and 5.9% (3) Hb S-β thalassaemia patients were on regular transfusions (> 8 transfusions/year). 26 (43.3%) patients were on HU therapy	Usage of HU was not consistent and the practice of transfusions was very variable among Sri Lankan patients with SCD
Barma et al. [60]	A prospective cohort study involving SCD children from Odisha, India	HU treatment significantly reduced the requirement of blood transfusion (5.4 U/Y to 2.4 U/Y and VOC (*p* < 0.001). Transfusion rate increased significantly (*p* < 0.001) among those who were not on HU (5.21 U/Y to 5.94 U/Y)	HU therapy could significantly reduce transfusion requirement and VOC among SCD children. Transfusion requirement under no HU therapy may increase with advancing age

Indications for HU therapy have been elucidated in a previously published evidence-based review in which authors (Wong et al.) suggested 8 recommendations in a graded system for HU therapy among patients with SCD of all ages [61]. In the present study, we analysed the practice of recommendations made by Wong et al., among South Asian patients. Five studies practiced HU therapy in accordance with first recommendation (Grade 1A) which is the usage of HU when adult Sickle cell anaemia (SCA) patients’ experience ≥ 3 moderate to severe pain crises in a 12-month period [29–31, 52, 53]. Seven studies practiced HU therapy in accordance with 2^nd^, 3^rd^ and 4^th^ recommendations (Grade 1 B) suggesting the usage of HU when adult SCA patient has a history of ACS or symptomatic anaemia; children with SCA experience ≥ 3 moderate to severe pain crises in 12-month time period or having a history of ACS or symptomatic anaemia [29–32, 34, 35, 57]. In addition, five studies practiced HU therapy in accordance with recommendation 6 (usage of HU in SCA patients who have a history of stroke) [29, 30, 34, 35, 57], and two studies [29, 42] in accordance with recommendation 7 (usage of HU in adult Hb S-β+ thalassaemia patients who experience ≥ 3 pain crises in 12-month period or having a history of ACS). Incidentally, 2 further studies reported the usage of HU in Hb S-D Punjab patients when they experience 3 or more VOC within 12-month period time [38, 40].

Of the 41 selected articles, only 9 reported the indications for transfusion therapy in sickle patients. Severe anaemia (Hb level < 6–7 g/dl) was the commonest indicator (n = 8) for transfusion therapy followed by VOC (n = 2), stroke (n = 1), splenic sequestration (n = 1), pregnancy (n = 1) and headache (n = 1). Wide range of pre-transfusion Hb levels were recorded in 2 studies. A study done in Central India reported a pre-transfusion Hb range which varied from 1.6 to 8.2 g/dl whereas another study from Gujarat reported a pre-transfusion Hb range which varied from 2 to 10 g/dl [36, 59].

We attempted to assess issues relating to demand and availability of hydroxyurea and blood transfusion in the region. We were unable to find reliable information on hydroxyurea. Blood transfusion services are organised differently and the adequacy of blood donation and the percentage of voluntary donors are variable in the different countries in the South Asian region. The state has total control over the blood banks in Sri Lanka Maldives and Bhutan’s while in India, Pakistan, Bangladesh and Nepal blood banking is heavily reliant on Non-Governmental organizations (NGO) and private blood banks though the state blood banks too exist. Overall, the demand for blood is not met in any of the countries except in Sri Lanka (Table 2). In 2017 India had the greatest absolute unmet blood unit requirement (40 964 075 units) from amongst 119 countries in the world [62].

**Table 2:** Estimated demand to supply ratio of blood in South Asian countries in 2017 according to the predictive model proposed by Nicholas Roberts and colleagues [62]

Country	Contributors of BTS	Demand to supply ratio^a^ according to Roberts et.al	Status remark
India	State, Private and NGO	3.0–5.0	Unmet demand
Pakistan	State, Private and NGO	1.0–2.0	Unmet demand
Sri Lanka	State	0.3–1.0	No unmet demand
Bangladesh	State, Private and NGO	5.0–10.0	Unmet demand
Bhutan	State	2.0–3.0	Unmet demand
Nepal	State, Private and NGO	2.0–3.0	Unmet demand
Maldives	State	1.0–2.0	Unmet demand

^a^Ratio of less than one indicates sufficient blood supply to meet the demand whereas ratio of more than one indicates unmet demand of blood

## Discussion

This systematic review of studies that evaluated the availability and therapeutic usage of both transfusion therapy and HU therapy for SCD in South Asia for the past 15 years identified that the available information in literature is limited and heterogeneous in nature. This precluded any effort of a proper meta-analysis. Even though presence of sickle haemoglobin had been reported from all South Asian countries, detailed studies of clinical outcomes were mostly available only from India. In most instances literature was restricted to case studies or case reports. One reason for this paucity of data could be the low prevalence of sickle haemoglobin in some countries in South Asia. For instance, studies from Sri Lanka and Bangladesh have shown that the prevalence of sickle haemoglobin was relatively lower than that of other haemoglobinopathies in these regions [63–65]. Sickle haemoglobin has been reported at comparatively higher prevalence from the Tharu community of Western Nepal and Pakistan [66, 67]. Although, the burden of the SCD in Tharu population had been acknowledged [68], no information was available of any evidence-based therapeutic strategy for patients with SCD from Nepal. The situation in Pakistan was not much different to that from Nepal. Other than the reports indicating the presence of SCD in Khyber Pakhtunkhwa, Karachi and Balochistan [69–71], nothing much is known about the clinical course of the disease and therapeutic scenarios currently in place for patients with SCD from these areas.

Even with limited data, the present review identified several indications for HU therapy for patients with SCD in India including, ≥ 3 pain crises/year, history of ACS, stroke and symptomatic severe anaemia. Nevertheless, in real world practice the circumstances could be quite different as explained by Jain et al.; in Maharashtra many patients with SCD have undergone HU therapy from the first clinical visit irrespective of their symptoms [47]. Moreover, the usage of HU for infants and children age 9 months or older who are asymptomatic or having infrequent pain episode has not adequately analysed among Indian patients. Therapeutic usage of HU for SCD have also been noted in couple of case reports from Pakistan, Bangladesh, Nepal and one descriptive cross-sectional study from Sri Lanka [21, 22, 26, 28]. However, particulars of different dosing regimens in practice, toxicities and detailed response to HU therapy is largely unknown. Despite all the known benefits of HU improving the quality of life, clinicians’ prescription and patient compliance of HU seems below par in the Indian subcontinent. In their recent review Jain and Mohanty described that the poor compliance with HU among Indian patients may be due to physician’s concerns of potential long-term mutagenic effects and lack of familiarity of primary attending medical staff with HU therapy [54]. Inconsistency in adherence with HU therapy owing to the lack of familiarity of primary care medical staff has also been noted in a recent Sri Lankan study in which authors recommended the development of national guidelines for management of patients with SCD [21]. Socio-economic status and the financial capabilities of the sickle patients largely influence the affordability of standard care including HU in economically disadvantaged settings in the Indian sub-continent. Nevertheless, country wise data and statistics about the availability and affordability of HU for sickle patients are not available in South Asia. Incidentally, initiations have been taken to deliver comprehensive care including free outpatient medication such as HU and pneumococcal vaccination for economically disadvantaged rural SCD communities in India with encouraging outcomes [41, 56]. However, no evidence is available about such initiatives outside India in South Asia.

Recent trials assessing the role of HU in preventing primary overt ischaemic stroke in patients with SCD of African origin found it to be effective [72, 73]. However, no such information is available about the efficiency of HU therapy in averting primary overt ischaemic stroke in patients with SCD of Indian origin.

Transfusion therapy for SCD has been used for many years and appears to be effective in primary and secondary prevention of stroke among sickle patients [74, 75]. The present review identified that transfusion therapy is in use for SCD in South Asia though there was paucity of information from outside India. However, indications of transfusion therapy have been described in limited number of studies. Available data suggests that transfusion was mostly given for severe anaemia (haemoglobin level below 6–7 g/dl). In addition, couple of Indian studies reported that transfusions were given when patients experience VOC [34, 38] and headaches [33]. Nevertheless, transfusion therapy for SCD seemed to be widely used in India without clear indications which could inevitably result in many deleterious clinical outcomes in patients and increase the financial burden. A study from Gujarat reported numerous transfusions for sickle patients with no clear diagnosis and justification [36]. Discrepancies and inconsistencies in transfusion practice for SCD has also been noted in Sri Lanka in which authors highlighted the disadvantage of not having a clear guideline [21]. Demand of blood for SCD transfusions is increasing in India by 0.99 million units per year. In line with projections by 2026 the total blood requirement for patients with SCD would reach 9.24 million units which would account for considerable portion of the total amount of donated blood [58]. There is however no data relating to blood requirements for patients with SCD outside India in the South Asian region.

## Conclusions

In summary, both HU and transfusion therapy for South Asian patients with SCD would benefit more from further evidence-based assessments and interventions. Fixed-low dose HU therapy (10 mg/kg/day), which has yielded promising results among Indian patients with SCD may be applicable for sickle patients with Indian origin from other South Asian countries. Similarly, the role of transfusion therapy for SCD should be well defined in different sickle communities of Indian subcontinent. Initiation has been taken by India introducing “National Health Mission Guidelines on Haemoglobinopathies” which included basic guidance on HU and transfusion therapy for SCD [76]. There is a clear gap of knowledge about the nature of SCD in the Indian subcontinent particularly from countries outside India. Practice of the main therapeutic modalities such as transfusion and HU therapies, diagnosis and different patient management strategies of SCD have not been adequately described in these regions, suggesting the compelling need for more research and evidence-based policy making.

## Data Availability

Data sharing not applicable to this article as no datasets were generated or analysed during the current study.

## Abbreviations

SCD: Sickle cell disease
Hb F: Foetal haemoglobin
HU: Hydroxyurea
TCD: Trans Cranial Doppler
Hb S: Sickle haemoglobin
VOC: Vaso-occlusive crisis
ACS: Acute chest syndrome
SCA: Sickle cell anaemia
NGO: Non-Governmental Organization
Hb SS: Homozygous sickle
